# A Study Examining the Functional Outcomes of Conservative and Surgical Management of Three- and Four-Part Proximal Humerus Fractures in Individuals Aged Over 50 Years

**DOI:** 10.7759/cureus.70368

**Published:** 2024-09-28

**Authors:** Manjunatha R, Naveena H M, Ankur Salwan, Ajay Koushik

**Affiliations:** 1 Department of Orthopedics, M.S. Ramaiah Medical College, Bengaluru, IND; 2 Department of Orthopedics, Subbaiah Medical College and Hospital and Research Centre, Molecular Biology Virology Laboratory, Shivamogga, IND; 3 Department of Orthopedics, Jawaharlal Nehru Medical College, Datta Meghe Institute of Higher Education and Research, Wardha, IND

**Keywords:** conservative treatment, constant score, elderly, functional outcome, philos plate, proximal humerus fractures

## Abstract

Background: Proximal humerus fractures are common in elderly patients and are often associated with osteoporosis. The management of these fractures, particularly three-part and four-part fractures, remains controversial, with conservative and surgical treatments both employed. This study compared conservative and surgical management's functional outcomes in subjects over 50 years.

Methods: This prospective observational study was conducted at Father Muller Medical College from December 2018 to June 2020. A total of 48 patients aged over 50 years with three-part and four-part proximal humerus fractures were included. Patients were divided into two groups: group A (conservative treatment with U-slab immobilization) and group B (surgical treatment with proximal humerus internal locking system plate fixation). Functional outcomes were assessed using the constant score at one-, three-, and 6-month intervals.

Results: Of the 48 patients, 25 (52.1%) had three-part fractures, and 23 (47.9%) had four-part fractures. At six months, the mean constant score for conservatively managed patients was significantly higher in both three-part fractures (77.23 vs. 52.58, p < 0.001) and four-part fractures (75.73 vs. 53.58, p < 0.001) compared to the surgically managed group. The conservative group also demonstrated better pain relief, range of motion, and shoulder strength. Complications were more common in the surgical group, with two cases of surgical site infection and one case of wound dehiscence, while no complications were observed in the conservative group.

Conclusion: Conservative treatment of three-part and four-part proximal humerus fractures in patients over 50 years provides better functional outcomes than surgical intervention. Conservative management should be considered the preferred treatment approach, especially in elderly patients with low-demand lifestyles.

## Introduction

Proximal humerus fractures are among the most common fractures in the elderly population, particularly in individuals over the age of 50, where osteoporosis is a prevalent underlying condition. These fractures account for approximately 5% of all fractures, with their incidence notably increasing with advancing age, often due to decreased bone density and increased susceptibility to low-energy trauma, such as falls from standing height [1,2]. The proximal humerus, particularly in osteoporotic patients, is prone to fracture due to the reduced structural integrity of the bone, making even minor falls a significant risk factor for injury [3]. The management of proximal humerus fractures remains a subject of debate, especially for more complex fracture patterns, such as three- and four-part fractures, which are classified based on the number of displaced bone segments [4]. These fracture patterns often present challenges due to the involvement of multiple fragments, which can disrupt the anatomical structure and function of the shoulder. Treatment options generally fall into two categories: conservative management and surgical intervention. Both approaches are widely used, with proponents for each citing different benefits and risks depending on the patient's condition, functional demands, and overall health status [5].

Conservative management, involving immobilization (commonly using a U-slab or other types of slings), followed by gradual physiotherapy, is often recommended for elderly patients with lower functional demands. This method relies on the body's natural healing process without surgical interference and aims to maintain functional mobility through controlled rehabilitation. The primary benefits of conservative management include the avoidance of surgical risks such as infection, neurovascular damage, and complications related to hardware implantation [6]. However, conservative treatment is not without challenges. Patients undergoing nonoperative care may experience stiffness, prolonged immobilization, and incomplete recovery of shoulder function, especially in complex fracture patterns [7].

On the other hand, surgical management is frequently employed in cases where anatomical alignment is significantly disrupted or in patients with higher functional demands requiring faster recovery. Techniques such as open reduction and internal fixation (ORIF) using devices like the proximal humerus internal locking system (PHILOS) plate are commonly used in the surgical treatment of three- and four-part proximal humerus fractures. The aim is to restore the anatomical alignment of the bone fragments, facilitate early mobilization, and ultimately achieve better functional outcomes [8]. However, surgery in elderly patients with osteoporosis poses unique risks. Poor bone quality can complicate fixation, leading to higher implant failure rates, avascular necrosis, and other complications such as infection or wound dehiscence [9,10]. Despite the theoretical advantages of surgical intervention in achieving anatomical realignment, recent studies have raised questions about whether surgery truly provides superior functional outcomes compared to conservative management, particularly in elderly patients with low-demand lifestyles. Several investigations have demonstrated that conservative treatment may yield comparable, or even better, functional outcomes in these patients, especially when assessed using validated scoring systems like the constant score, which evaluates pain, daily activities, range of motion (ROM), and muscle strength [11]. For example, research has shown that elderly patients treated conservatively often experience fewer complications, better pain relief, and improved functional mobility than surgery [12,13].

This evolving perspective is particularly important given the demographic shift toward an aging population, where the incidence of proximal humerus fractures is expected to rise. As more elderly individuals present with these injuries, healthcare providers must carefully weigh the benefits and risks of surgical versus conservative treatment. Factors such as the patient's comorbidities, bone quality, functional demands, and personal preferences must all be considered to determine the most appropriate treatment plan [14]. The increasing body of evidence suggesting favorable outcomes with conservative management challenges the conventional preference for surgery in more complex fractures, prompting further investigation into which approach optimally balances functional recovery with patient safety. This study aimed to contribute to this ongoing discussion by comparing the functional outcomes of conservative and surgical management in patients aged over 50 years with three- and four-part proximal humerus fractures. The study assessed these outcomes using the constant score over a six-month follow-up period to comprehensively analyze recovery, pain, ROM, and complications in both treatment groups.

## Materials and methods

Study design

This prospective, observational study was conducted at the Department of Orthopedics, Father Muller Medical College, between December 2018 and June 2020. The primary objective was to compare the functional outcomes of conservative management versus surgical management in patients aged over 50 years with three- and four-part proximal humerus fractures. The study was designed to track patients' progress six months after treatment to evaluate which management strategy resulted in better functional recovery.

Study population

The study enrolled 48 patients who met the inclusion criteria. These patients were aged 50 years and above, with confirmed three- or four-part proximal humerus fractures according to Neer’s classification. All participants were selected from patients treated at the study center. Each patient was assessed for eligibility based on clinical examination and radiographic findings. Upon enrollment, patients were stratified into two groups based on their chosen treatment modality: group A consisted of patients who received conservative treatment, whereas group B included patients who underwent surgical management.

Inclusion criteria and exclusion criteria

Patients were included in the study if they were 50 or older and had sustained a Neer's three- or four-part proximal humerus fracture. Eligible patients had to consent to participate in the study and agree to regular follow-up for at least six months. The decision for either conservative or surgical management was made based on the patient's condition, the severity of the fracture, patient preference, and surgeon's discretion. The study excluded patients with open fractures, undisplaced fractures, or fractures associated with neurovascular injuries. Patients who had undergone previous shoulder surgeries were excluded; as such, interventions could influence functional outcomes. Additionally, patients with pathological fractures (e.g., due to malignancy), immunocompromised status, those on chronic steroid therapy, or those with significant comorbidities that could affect recovery were excluded. Finally, patients lost to follow-up during the six months were excluded from the final analysis to ensure data integrity.

Treatment protocols

Group A: Conservative Management

Patients in group A were treated conservatively using U-slab immobilization of the injured upper limb. The U-slab provided external stabilization for three weeks, during which patients were advised to limit movement to promote fracture healing. After three weeks, gradual limb mobilization was initiated per the patient's tolerance. Pendulum exercises were first introduced, followed by progressive ROM exercises to improve shoulder mobility and strength. Patients were monitored during this period for any signs of complications, such as stiffness or delayed fracture healing.

Group B: Surgical Management

Patients in group B underwent surgical intervention, specifically ORIF, using the PHILOS plate. The surgical procedure was performed under general anesthesia via the deltopectoral approach. This approach allowed for better access to the fracture site and ensured stable fixation using locking plates and screws designed to provide optimal fixation even in osteoporotic bone. Postoperatively, the limb was immobilized in a broad arm sling, and passive mobilization exercises were started in the second week. Pendulum exercises were followed by gradual increases in the ROM, emphasizing pain management and functional recovery. Patients were assessed for surgical site healing and potential complications, including infection, wound dehiscence, or hardware failure.

Follow-up and outcome assessment

Patients in both treatment groups were evaluated at regular intervals of one, three, and six months following the initiation of their respective treatments. The functional outcomes were systematically assessed during each visit using the constant score, a validated tool widely used for evaluating shoulder function. The constant score provides a comprehensive assessment based on four key domains: pain, ability to perform daily activities, ROM, and muscle strength. This score ranges from 0 to 100, where higher scores indicate better shoulder function and overall outcomes. Pain was one of the primary metrics assessed during the follow-up visits. It was evaluated using a 15-point scale, with patients self-reporting their level of pain [15]. This allowed for a subjective yet standardized assessment of the pain experienced during different stages of recovery. The ability to perform activities of daily living was the next domain assessed, using a 20-point scale. This scale evaluated the patient's capacity to engage in routine tasks, such as eating, dressing, and performing overhead activities, which are critical indicators of shoulder functionality and rehabilitation progress.

In addition to functional performance, the shoulder's ROM was measured during each follow-up visit. ROM was recorded in degrees for forward flexion, abduction, and external and internal rotation using a goniometer, a standard tool for measuring joint angles. Improved ROM is indicative of a patient's recovery, especially in terms of flexibility and mobility. Shoulder strength was another important parameter evaluated, focusing specifically on abduction strength. Patients were asked to maintain shoulder abduction at a 90° angle against resistance. Their ability to hold this position while resisting a measured weight was recorded in kilograms, objectively assessing muscle recovery and strength. At each follow-up, radiographic assessments were also conducted to monitor the progress of fracture healing. Radiographs allowed clinicians to identify potential complications, such as nonunion, avascular necrosis, or implant loosening, in patients who had undergone surgical management. These assessments provided valuable insights into the healing bone's structural integrity and surgical implants' stability. Any adverse symptoms reported by the patients, including pain, swelling, or restricted movement, were recorded, and any complications encountered during the follow-up period were documented to ensure a comprehensive evaluation of both treatment modalities.

Data collection and statistical analysis

Demographic data, including age, gender, type of fracture, and cause of injury, were collected at enrollment. Outcome data, including constant score parameters, complications, and radiological findings, were collected at each follow-up visit. Statistical analysis was performed using the SPSS software, version 23 (IBM Corp., Armonk, NY). Descriptive statistics, such as means and standard deviations, were calculated for continuous variables, whereas frequencies and percentages were used for categorical variables. As measured by the constant score, functional outcomes were compared between the two groups at each time point using a repeated-measures analysis of variance. The chi-square test assessed differences in categorical variables, such as complications between the conservative and surgical groups. A p value of less than 0.05 was considered statistically significant, indicating meaningful differences in the functional outcomes between the two treatment modalities.

Ethical considerations

The Institutional Ethics Committee of Father Muller Medical College FMMCIEC/CCM/507/2018 reviewed and approved the study protocol. Written informed consent was obtained from all patients before they participated in the study. All patient data were anonymized to maintain confidentiality, and the study adhered to the ethical principles outlined in the Declaration of Helsinki.

## Results

Table 1 shows that the study included 48 patients, with 20 men and 28 women. Most patients (N = 27, 56.2%) were between 51 and 60 years old.

**Table 1:** Demographic characteristics of the study population SD: standard deviation

Parameter	N (%)	Mean (SD)
Age (years)		
51-60 years	27 (56.2%)	61.27 (8.5)
61-70 years	15 (31.3%)	
>70 years	6 (12.5%)	
Gender		
Male	20 (41.7%)	
Female	28 (58.3%)	

Table 2 shows that among the 48 patients, 25 (52.1%) had three-part fractures, whereas 23 (47.9%) had four-part fractures. The most common cause of injury was slip and fall, reported in 38 patients (79.1%).

**Table 2:** Distribution of fracture type and injury cause

Parameter	N (%)
Three-part fractures	25 (52.1%)
Four-part fractures	23 (47.9%)
Slip and fall injury	38 (79.1%)
Road traffic accidents	10 (20.8%)

Table 3 shows that the constant score was used to evaluate functional outcomes. The mean constant score at the six-month follow-up for three-part fractures was 77.23 (conservative) and 52.58 (surgical), whereas for four-part fractures, it was 75.73 (conservative) and 53.58 (surgical). Overall, patients treated conservatively had significantly better outcomes.

**Table 3:** Functional outcome comparison: constant score at six months

Fracture type	Treatment	N	Mean constant score (six months)	p value
Three-part	Conservative	13	77.23	<0.001
	Surgical	12	52.58	
Four part	Conservative	11	75.73	<0.001
	Surgical	12	53.58	
Overall	Conservative	24	76.54	<0.001
	Surgical	24	53.08	

Table 4 compares pain scores between the two groups over six months. At one month, the two groups had no statistically significant difference in pain. However, the conservative group had significantly better pain scores by three and six months.

**Table 4:** Pain score comparison between the surgical and conservative treatments

Follow-up duration	Treatment	N	Mean pain score	p value
One month	Surgical	12	4.58	0.846
	Conservative	13	4.23	
Three months	Surgical	12	5.42	0.001
	Conservative	13	10.77	
Six months	Surgical	12	7.92	<0.001
	Conservative	13	14.62	

Table 5 shows that ROM was assessed at each follow-up. At six months, the conservative group had significantly better shoulder abduction, forward flexion, and internal rotation outcomes.

**Table 5:** ROM comparison at six months ROM: range of motion

Follow-up duration	Treatment	N	Mean ROM (degrees)	p value
Abduction	Surgical	12	80	<0.001
	Conservative	13	100	
Forward flexion	Surgical	12	90	<0.001
	Conservative	13	112	
Internal rotation	Surgical	12	Lumbosacral junction	<0.001
	Conservative	13	Lumbosacral junction	

Table 6 shows complications were noted in three patients (6.25%) from the surgical group, including two cases (4.2%) of surgical site infections and one case (2.1%) of wound dehiscence. No complications were observed in the conservative group.

**Table 6:** Complications observed in the study population

Complication	N (%)
Surgical site infection	2 (4.2%)
Wound dehiscence	1 (2.1%)
Total complications (surgical group)	3 (6.25%)

## Discussion

The management of proximal humerus fractures, particularly in elderly patients with osteoporosis, remains a challenging clinical decision. This study compared the functional outcomes of conservative versus surgical management of three- and four-part proximal humerus fractures in subjects aged over 50 years. Our findings demonstrate that conservative treatment resulted in superior functional outcomes at six months compared to surgical intervention, as measured by the constant score. This aligns with prior studies that have suggested that nonoperative management of such fractures can provide favorable outcomes, especially in elderly patients with lower physical demands and poor bone quality [4,9].

Our study showed that patients in the conservative group had significantly better pain relief, ROM, and muscle strength compared to the surgically managed group. At six months, the mean constant scores were notably higher for three- and four-part fractures in the conservative group. This is consistent with findings from similar studies that emphasize the benefits of nonoperative management for improving shoulder functionality in elderly populations [1-3]. These results underscore the importance of tailoring treatment plans based on individual patient characteristics, such as age, activity level, and bone health [10].

The surgical group in our study experienced a higher incidence of complications, including two cases of surgical site infection and one case of wound dehiscence. Previous studies have also reported higher complication rates associated with surgical interventions for proximal humerus fractures in elderly patients, particularly due to poor bone quality and the technical challenges of internal fixation [16,17]. Furthermore, the risk of implant failure, nonunion, and avascular necrosis remains a concern with surgical management, as highlighted by recent literature [18]. While the PHILOS plate provides stable fixation, especially in osteoporotic bone, its use in elderly patients should be approached with caution due to the increased risk of complications [19].

The limitations of our study should be acknowledged. First, the relatively small sample size may limit the generalizability of our findings. While the observed differences in functional outcomes are statistically significant, larger studies are needed to confirm these results in more diverse populations. Second, the follow-up period was limited to six months, which may not capture long-term complications or functional decline, such as osteonecrosis or implant-related issues. Longer term follow-up is essential to determine whether the early advantages of conservative management persist over time. Finally, the nonrandomized nature of the study introduces potential selection bias, as both surgeon and patient preferences influenced the choice of treatment [20]. Future randomized controlled trials are needed to provide more definitive evidence on the optimal management strategy for proximal humerus fractures in elderly patients.

Limitations

One limitation of this study is the relatively small sample size, which may limit the generalizability of the results to a broader population. Additionally, the study was conducted at a single tertiary care center, which could introduce bias based on local practices or surgeon expertise, potentially affecting the outcomes of surgical management. The follow-up period was limited to six months, which may not capture long-term functional outcomes or complications such as osteonecrosis, implant failure, or late-onset shoulder stiffness. Another limitation is the lack of randomization, as the choice of treatment was influenced by the surgeon's preference and patient factors, which could lead to selection bias. Furthermore, the study did not assess the impact of patient comorbidities on the recovery process, which may play a significant role in functional outcomes, especially in the elderly population. Finally, while the constant score assessed functional outcomes, it may not fully capture all aspects of patient satisfaction and quality of life after treatment.

## Conclusions

In conclusion, managing three- and four-part proximal humerus fractures in patients over 50 presents significant challenges, especially in the context of osteoporosis and reduced bone quality. This study demonstrates that conservative treatment yields better functional outcomes than surgical intervention, as evidenced by higher constant scores, improved pain relief, ROM, and shoulder strength. Additionally, conservative management was associated with fewer complications. Given these findings, conservative treatment should be preferable, particularly for elderly patients with lower physical demands. However, the treatment choice must be individualized, considering the patient's overall health, functional goals, and the surgeon's expertise. Further studies with larger sample sizes and longer follow-up periods are needed to validate these findings and assess long-term outcomes.
